# Prediction of bone oligometastases in breast cancer using models based on deep learning radiomics of PET/CT imaging

**DOI:** 10.3389/fonc.2025.1621677

**Published:** 2025-08-21

**Authors:** Guoxiu Lu, Ronghui Tian, Wei Yang, Jiayi Zhao, Wenjing Chen, Zijie Xiang, Shanhu Hao, Guoxu Zhang

**Affiliations:** ^1^ College of Medicine and Biological Information Engineering, Northeastern University, Shenyang, Liaoning, China; ^2^ Department of Nuclear Medicine, General Hospital of Northern Theater Command, Shenyang, Liaoning, China; ^3^ School of Software, Shenyang University of Technology, Shenyang, Liaoning, China; ^4^ Department of Radiology, Cancer Hospital of China Medical University, Liaoning Cancer Hospital and Institute, Shenyang, Liaoning, China; ^5^ Department of Nuclear Medicine, Dalian Medical University, Dalian, China; ^6^ Department of Research and Development, United Imaging Intelligence (Beijing) Co., Ltd., Beijing, China; ^7^ Biomedical Engineering, Shenyang University of Technology, Shenyang, Liaoning, China

**Keywords:** breast cancer, bone oligometastases, deep learning, radiomics, deep learning radiomics, PET/CT

## Abstract

**Objective:**

To develop a deep learning radiomics(DLR)model integrating PET/CT radiomics, deep learning features, and clinical parameters for early prediction of bone oligometastases (≤5 lesions) in breast cancer.

**Methods:**

We retrospectively analyzed 207 breast cancer patients with 312 bone lesions, comprising 107 benign and 205 malignant lesions, including 89 lesions with confirmed bone metastases. Radiomic features were extracted from computed tomography (CT), positron emission tomography (PET), and fused PET/CT images using PyRadiomics embedded in the uAI Research Portal. Standardized feature extraction and feature selection were performed using the Least Absolute Shrinkage and Selection Operator (LASSO) method. We developed and validated three models: a radiomics-based model, a deep learning model using BasicNet, and a deep learning radiomics (DLR) model incorporating clinical and metabolic parameters. Model performance was assessed using the area under the receiver operating characteristic curve (AUC), accuracy, sensitivity, and specificity. Statistical comparisons were conducted using the DeLong test.

**Results:**

Visual assessment of fused PET/CT images identified 227 (72.8%) abnormal lesions, demonstrating greater sensitivity than CT or PET alone. The complex radiomics model achieved a sensitivity of 98.9% [96.1%–99.4%], specificity of 98.2% [88.1%–99.6%], accuracy of 98.7% [89.6%–99.5%], and area under the curve (AUC) of 0.989. The BasicNet model outperformed other transfer learning models, achieving an AUC of 0.961. The DeLong test confirmed that the AUC of the BasicNet model was significantly higher than the traditional radiomics model. The DLR+Complex model with a random forest classifier achieved the highest overall performance, with an AUC of 0.990, sensitivity of 98.6%, specificity of 90.5%, and accuracy of 99.8%.

**Conclusions:**

The BasicNet model significantly outperformed traditional radiomics approaches in predicting bone oligometastases in breast cancer patients. The DLR+Complex model demonstrated the best predictive performance across all metrics. Future strategies for precise diagnosis and treatment should incorporate histologic subtype, advanced imaging, and molecular biomarkers.

## 1 Introduction

Breast cancer is among the most prevalent malignant tumors globally and remains a major health concern for the female population (1).However, approximately 50%–70% of locally advanced breast cancer cases eventually metastasize to distant organs such as the lungs, liver, and bones (2). Globally, bone metastases represent a leading cause of death from breast cancer, occurring in 30%–70% of patients with advanced breast cancer, accounting for approximately 500,000 new global cases annually. Bone metastases from breast cancer remain a serious condition, contributing significantly to patients’ morbidity and mortality (3). Patients with bone-only metastases generally exhibit longer overall survival compared to those with widespread metastases involving visceral organs such as the liver and lungs. And the later patients have a median survival of only 24–36 months (4).One prior study also found that patients with bone metastases had a better survival rate than those with visceral metastases, with a 5-year survival rate of up to 20% and a median survival time of more than 72 months in some patients, indicating that a large proportion of breast cancer bone metastases were in a state of oligometastases with inert biological behavior (5). Current definitions of bone oligometastases vary across studies, While some trials define it as ≤5 lesions (4, 6–8), others include up to 3 lesions (9). This study we adopts the definition as five or fewer metastatic lesions confined to the skeletal system pathologically proven by bone scan or PET-CT, and the lesions could be considered as having oligometastases.

Early recognition and accurate diagnosis of bone oligometastases metastases with breast cancer are critical to their further treatment.Advances in medical technology and increased awareness have significantly improved the early detection and treatment of the disease (10). Traditional imaging modalities such as bone scintigraphy, computed tomography (CT), and magnetic resonance imaging (MRI) are commonly used to assess metastatic bone disease (11–13). However, these techniques have notable limitations. For example, the sensitivity, specificity, positive predictive value, and negative predictive value of 99mTc-MDP bone scintigraphy for detecting skeletal metastases are only 67%, 78%, 50%, and 50%, respectively (14). Moreover, early micrometastases (<5 mm), particularly osteolytic lesions, are often missed by CT or scintigraphy, with missed diagnosis rates as high as 40% (15). Inter-observer variability also poses a challenge (Kappa values: 0.65–0.72), and single-modality imaging fails to fully capture tumor metabolic heterogeneity and molecular characteristics (16). Although PET/CT improves sensitivity (up to 94%) with 18F-FDG imaging, it involves higher radiation exposure (14–20 mSv), and its quantitative analysis of metabolic parameters (e.g., SUVmax slope) often depends on empirical thresholds (13, 17, 18). These limitations have driven research toward more advanced multimodal imaging strategies.

Recent progress in artificial intelligence and deep learning has transformed medical image analysis, particularly in the detection of metastatic breast cancer. Deep learning radiomics (DLR), which integrates features from multiple imaging modalities, has notably emerged as a promising approach. Ceranka et al. (19) previously developed a fully automated deep learning method for detecting and segmenting bone metastases on whole-body multiparametric MRI. Their system outperformed existing methods, achieving 63% sensitivity with a mean of 6.44 false positives per image and a Dice coefficient of 0.53. In another study, Shang et al. (20) enhanced sensitivity using a Multi-Perspective Extraction module in the feature extraction phase, utilizing three different sizes of convolutional kernels to enhance sensitivity to bone metastases. Their BMSMM-Net allowed high-performance segmentation of bone metastases, achieving F1 scores of 91.07% and 95.17% for segmenting bone metastases and bone regions, respectively, along with mIoU scores of 83.60% and 90.78%.

^18^F-FDG PET/CT imaging is commonly used in diagnosis and follow-up of metastatic in breast cancer, but its quantitative analysis is complicated by the number and location heterogeneity of metastatic lesions. In some studies, by combining MRI, CT, and PET imaging data, researchers were able to more fully assess the risk of bone metastases in breast cancer. Moreua (21) proposed a completely automatic deep learning based method to detect and segment bones and bone lesions with 24 patients on ^18^F -FDG PET/CT in the context of metastatic in breast cancer, and they introduced an automatic PET bone index which could be incorporated in the monitoring and decision process. Moreua (22) also proposed networks to segment breast cancer metastatic lesions on longitudinal whole-body PET/CT with 60 patients and extract imaging biomarkers from the segmentations and evaluate their potential to determine treatment response. Their works constituted promising tools for the automatic segmentation of lesions in 60 patients with metastatic breast cancer allowing treatment response assessment with several biomarkers.

DLR offers a new paradigm for the accurate diagnosis of bone oligometastases. By extracting high-throughput features from PET/CT—including textural features (e.g., Gray Level Co-occurrence Matrix (GLCM) entropy, GLSZM regional variance) and morphological characteristics (e.g., sphericity)—DLR enables quantification of tumor heterogeneity and risk prediction. However, few studies have applied DLR specifically to bone oligometastases in breast cancer. Such models enhance multimodal feature integration and reduce overfitting. This study aims to develop a DLR model integrating PET/CT radiomics, deep learning features, and clinical parameters for early prediction of bone oligometastases (≤5 lesions) in breast cancer.

## 2 Materials and methods

### 2.1 Patient population

This retrospective study was approved by the institutional review board of our hospital (Approval No. Y (2025)-022), who further waived the requirement for informed consent. A total of 10,893 female patients who underwent PET/CT examinations at our centre between January 2012 and January 2025 were reviewed. Clinical data were obtained from our hospital’s electronic medical record system, including pathology reports, laboratory tests, and treatment records. Imaging data included original PET/CT DICOM images and structured departmental reports.

Inclusion criteria were:

1. Pathological biopsy-confirmed primary breast cancer.
2. Age between 18 and 75 years.
3. Underwent baseline PET/CT performed before treatment.
4. Confirmed as having bone lesions, defined as ≤5 bone lesions, confirmed via pathology (≥1 lesion) or follow-up imaging (CT/MRI) for at least 6 months.
5. Complete imaging and clinical data available.

Exclusion criteria included:

1. History of other malignancies.
2. Poor image quality due to motion artifacts or equipment failure.
3. Prior radiotherapy or surgery for bone metastases.
4. More than five bone lesions.
5. Absence of pathological confirmation.

**Figure 1:**
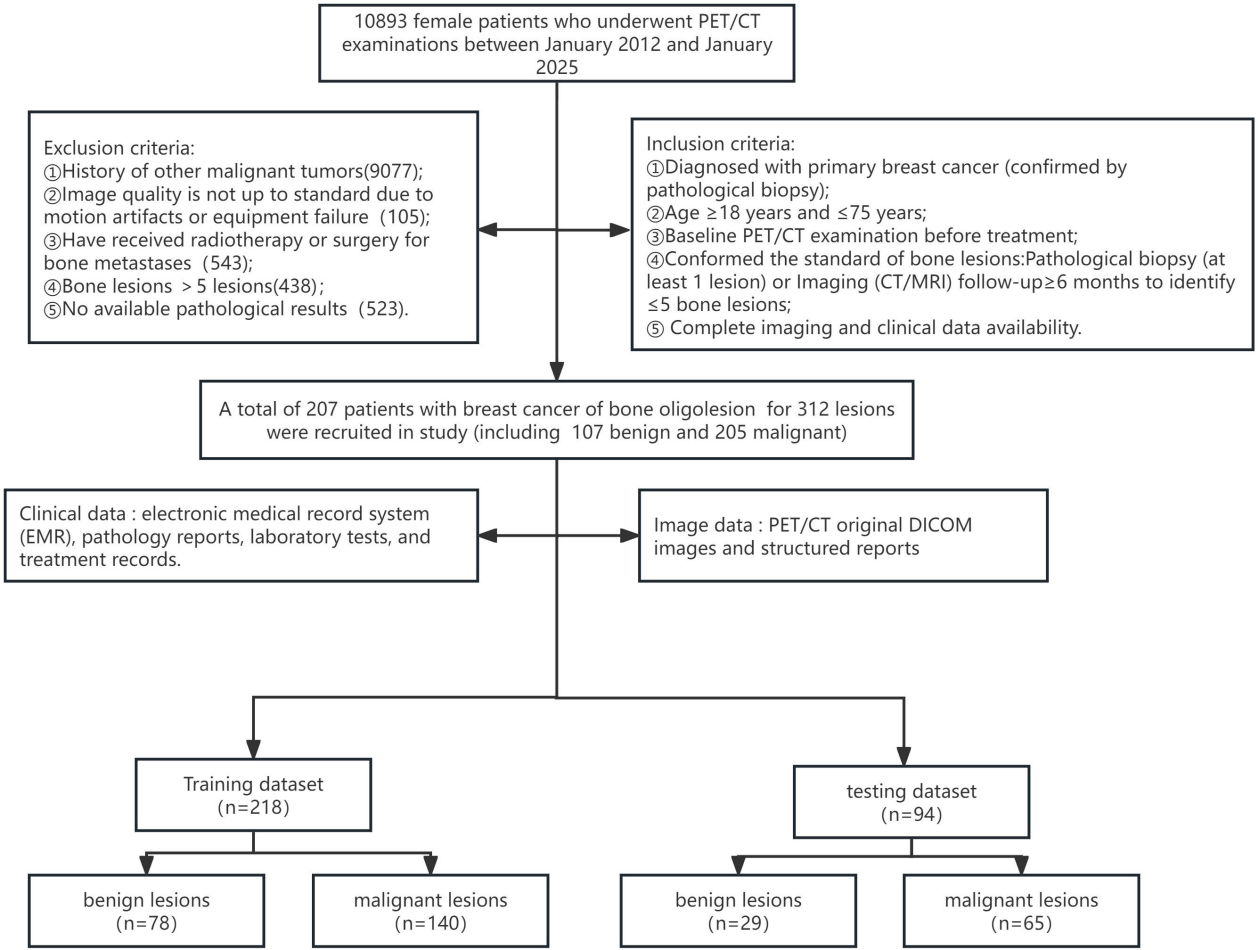
Flowchart of patient recruitment.

Ultimately, 207 patients with 312 breast tumor-associated bone lesions were included. Of these, 107 lesions were benign and 205 were malignant. The dataset was split into a training and testing cohort in a 7:3 ratio. The training cohort consisted of 218 lesions (78 benign, 140 malignant), while the internal testing cohort included 94 lesions (29 benign, 65 malignant). The enrollment process is outlined in **Figure 1**.

### 2.2 Image acquisition

Images were acquired using the GE Discovery VCT and GE Discovery 710 PET/CT scanners. ^18^F-FDG was synthesized on a GE Minitrace cyclotron, with a radiochemical purity >98%. Patients fasted for more than 6 hours before the scan, with blood glucose controlled below 6.1 mmol/L. ^18^F-FDG was intravenously administered at a dose of 5.5 MBq/kg, and imaging was performed 40–60 minutes post-injection.

The imaging protocol included a non-contrast CT scan, followed by a PET scan, covering from the mid-femur to the skull vertex, and including the lower limbs when necessary. CT parameters were: 120 kV, 110 mA, pitch 1.0; rotation time 0.5 s; and slice thickness, 3.27 mm. PET images were collected in the same range, with a body collection time of 2–3 min/bed. In total, 6–8 beds were collected by 3D PET scanning, with each bed taking 1.5min. The computer system automatically performed image reconstruction using Ordered Subsets Expectation Maximization for coronal, sagittal, and transverse views and 3D projections.

### 2.3 Region of interest segmentation

Bone oligolesions of breast cancer were selected for region of interest (ROI) segmentation on the largest layer of the tumor. ROI segmentation in PET/CT images is a critical step in deep learning and image-based data analysis. To ensure the consistency and reliability of the data, we applied standardized image segmentation. All PET/CT DICOM images were imported into 3D Slicer (version 5.2) (23) and uploaded to the uAI Research Portal (version 20241130) in both DICOM and nii.gz formats for deep learning and radiomics analysis. Lesions were identified on CT by the presence of lytic or sclerotic changes, with or without associated soft tissue mass, abnormal postcontrast enhancement. In PET, the maximum standard ingested value (SUVmax) was calculated using both visual and semi-quantitative methods. Abnormal lesions were defined as those with an increased FDG uptake, with an SUVmax higher than physiologic hepatic background activity.

Three experienced readers (two radiologists and one nuclear medicine physician with 10–12 years of experience each) independently delineated each ROI along the tumor margin, from the first to the last layer of the whole tumor, using 3D Slicer, under the supervision of a senior radiologist (30 years’ experience). All readers were blinded to histopathological results. We traced abnormal areas in these images and attempted to delineate the burr at the edge of each tumor.

The ROI segmentation followed strict criterias:

1. Include the entire lesion as completely as possible.
2. Minimize inclusion of surrounding non-lesion tissue.
3. For lesions with unclear boundaries, integrate PET and CT data for delineation.
4. Necrotic regions(Areas of necrosis in bone metastases are areas within the bone metastases that have formed due to tumor cell death and destruction of tissue structure due to a variety of reasons)or calcified regions(Calcified areas in bone metastases are areas of higher density within or around bone metastases that are formed as a result of abnormal local deposition of calcium salts due to the metabolism and proliferation of tumor cells as well as the body’s repair processes)were included for accurate radiomic feature extraction.Necrotic/calcified regions were included if occupying >10% of lesion volume (verified by 3D Slicer’s volumetric analysis) to ensure radiomic feature stability.

**Figure 2:**
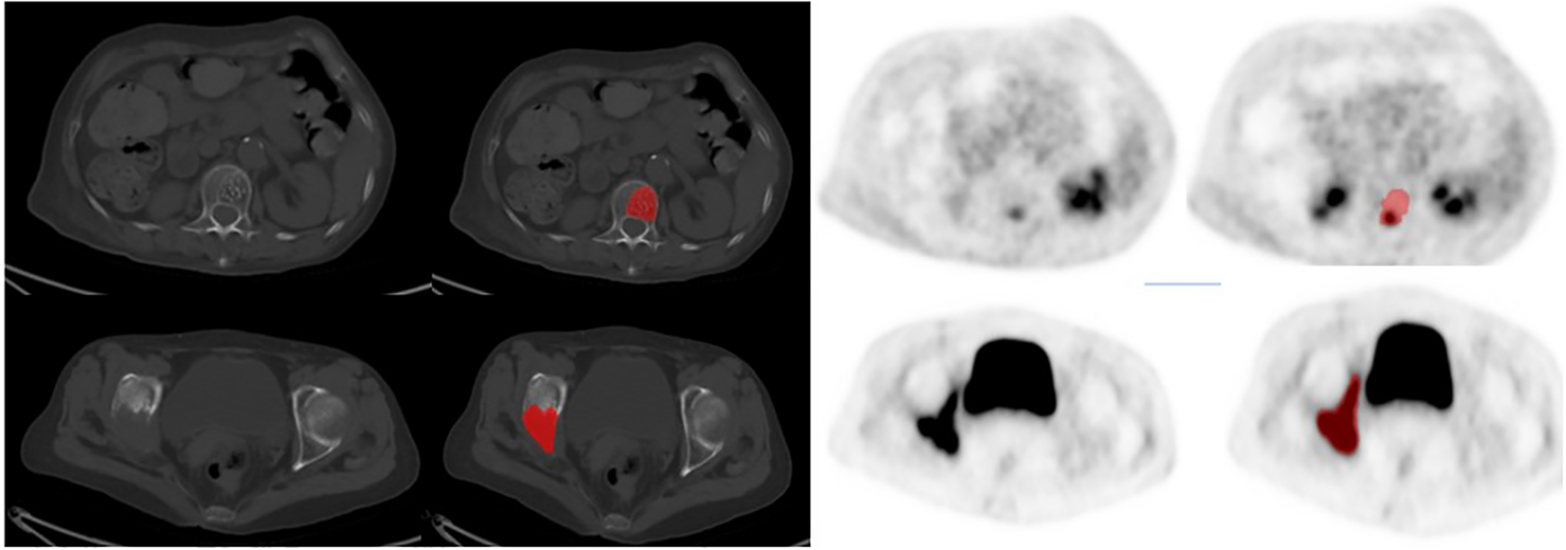
The typical imagings of two patients:The first row of images showed a 67-year-old woman, 6 months after right breast cancer surgery, CT showed the first lumbar had low density foci, and accompanied with multiple punctate high-density focals, PET images of this lesion local metabolism slightly increased, and finally diagnosed as benign hemangioma after 6 months of clinical follow-up. The second row of images showed a 72-year-old woman, 4 years after left breast cancer surgery, CT showed right sciatic and right iliac were all destructed, PET images of these lesions' metabolism were obviously increased, and finally diagnosed as bone metastasis with puncture pathology.

Each patient’s ROI segmentation took approximately 10–15 minutes, with a review time of 5–8 minutes. Example segmentations are shown in **Figure 2**.

### 2.4 Model Selection and Transfer Learning

We selected a 3D deep supervised residual BasicNet model (24) for transfer learning, due to its efficiency and proven effectiveness in medical image analysis. BasicNet, a lightweight convolutional neural network architecture with moderate number of parameters, high computational efficiency, and ease of migration, is particularly suitable for lesion detection and classification tasks in medical imaging. Compared to ResNet-101 (25) (23.5M params) and 3D U-Net (19.7M) (26), BasicNet’s lightweight design (4.2M params) achieved faster convergence (98% accuracy by epoch 50 vs. 65 in ResNet) with lower computational cost. This model was pre-trained on ImageNet for robust feature extraction and fine-tuned on PET/CT bone lesion data.

Separate transfer learning processes were implemented for PET and CT to leverage modality-specific information. The network comprised an encoder–decoder architecture adopted as the mainstream network, residual connections, and deep supervision layers. The parameter configuration during the training process had a decisive impact on the model performance. Optimal training parameter combinations were determined. Through many preliminary experiments and a literature research. Considering the common category imbalance problem in medical data, similar to the 3D U-Net (27), this study used the loss function to choose focal loss function (Focal Loss) instead of the traditional cross-entropy loss (28). Target weights were set to reflect the different tolerances for false positives and false negatives in clinical practice, with weights set to 0.3 for benign lesions and 0.7 for malignant lesions. This optimal combination was determined by iteratively adjusting the weight values through receiver operating characteristic (ROC) curve analysis and clinical expert assessment. Adaptive Moment Estimation was selected as the optimizer. Compared with the traditional stochastic gradient descent method, Adam was able to adaptively adjust the learning rate of each parameter, making the training process more stable and achieving more rapid convergence. The learning rate was initially set to 0.0001, but was gradually reduced during the training phase to find the global minimum of the loss function. The number of training iterations was set to 1001, and the batch size was set to 32 samples per batch, defined as a balance between model performance and computational resources. To fully utilize the parallel computing capability of the multi-core processor, the number of IO threads was set to 8, which significantly accelerated the data loading and preprocessing process and reduced the IO bottleneck in training.

For the training process, we adopted a validation set performance monitoring strategy, in which the model performance was evaluated on an independent validation set every 10 epochs. The accuracy, sensitivity, specificity, AUC value, and many other indicators were assessed. An early stopping mechanism was applied to prevent overfitting. The training process was automatically terminated when the validation set performance did not improve for five consecutive evaluations. At the end of training, the model parameters saved at the epoch point with the best performance on the validation set were collected. This strategy avoided overfitting which may otherwise occur in the late stage of training, and further ensured the generalization capabilities of the model.

The Max Pooling layer (29) was used to extract the most salient features in the image, retaining structural information while reducing data dimensionality and computational complexity. Specifically, the output of the penultimate maximum pooling layer was extracted, to retain sufficient semantic information, while ensuring a high spatial resolution.

We further adopted a pre-fusion (30) approach to obtain joint PET/CT features by fusing deep learning features from both CT and PET. This pre-fusion strategy achieved information integration at the feature level, preserving the semantic relevance of the original features, and capturing inter-modal interactions better than post-fusion (decision-level fusion). For concrete implementation, we first normalized the feature vectors of the two modalities, and then merged them into one augmented feature vector by concatenation operation.

### 2.5 Extraction of radiomics features

Radiomics feature extraction, in which PET/CT images are quantitatively analyzed to extract feature information difficult to recognize with the naked eye, is one of the core aspects of this study. In the present study, the PyRadiomics (31) embedded in the uAI Research Portal was applied for standardized feature extraction. Prior to feature extraction, images were first preprocessed, including voxel resampling to 1×1×1 mm³ to eliminate any specific differences in scanning parameters between different devices, while a standardized grayscale discretization algorithm was applied to quantify the grayscale values to a fixed number of bins. This was then used to segment and extract radiomic features from the semi-automated segmentation of attenuation-corrected PET images by selecting an absolute SUVmax threshold of 2.2. For PET images, the bin size was 0.7936508 and the number of gray levels in intensity discretization was 64; further, an absolute intensity rescaling with a minimum bound of zero and maximum of 50 was selected. For intensity discretization of CT images, the bin size was 32 and the number of gray levels was 400. Kernel 3 was applied, and an intensity rescaling of minimum bound of −1000 and maximum bound of 3000 was applied (32). A circular 3D ROI in the region with the highest FDG avid bone lesion was chosen for analysis. This preprocessing step ensured the stability and comparability of the extracted features.

Radiomics was predominantly conducted using the following stems: First-order feature, second order texture, conventional PET/CT parameters (SUV and TLG), shape feature, GLCM, Gray Level Run Length Matrix, GLRLM), Gray Level Size Zone Matrix (GLSZM), Gray Level Dependence Matrix (Gray Level Dependence Matrix, GLDM) and Neighboring Gray Tone Difference Matrix.

### 2.6 Feature selection and model construction

Feature selection and model construction sessions are key to ensure the performance of the final classifier. In the present study, we adopted a multi-step feature selection strategy combined with several machine learning algorithms to achieve the accurate qualitative diagnosis of bone oligo lesions. First, Z-score normalization was conducted on all extracted deep learning features and radiomics features to transform feature values into a standard distribution with a mean of 0 and a standard deviation of 1. To initially reduce the spatial dimensionality of the features, the Mann–Whitney U test (a nonparametric test) was applied to assess the ability of each feature to discriminate between groups of benign and malignant lesions. In terms of initial threshold setting, the Mann Whitney U test uses a relatively loose p<0.05 threshold to avoid premature exclusion of potentially useful features. We chose a nonparametric test rather than a t-test based on the consideration that medical imaging features generally do not conform to the normal distribution assumption. Indeed, there is often a high degree of correlation between medical imaging features, and this redundant information not only increases computational complexity, but may also lead to unstable model performance. Pearson correlation analysis was applied to calculate the correlation between feature parameters and construct the feature correlation matrix. Pearson correlation threshold (|r|>0.85) was selected to balance feature independence and information retention, validated by 10-fold CV showed optimal AUC at this threshold.Those showing a stronger correlation with the target variable (benign and malignant classification) were retained. The remaining features were further screened using LASSO, and the sparse representation of the features is achieved by introducing the L1 regularization term, which drives some of the regression coefficients to be precisely equal to zero. The present study used a 5-fold cross-validation method to find the optimal value among a series of candidate λ values. Experiments show that when λ is set to approximately 0.015, the model reached the optimal equilibrium point, and the screened subset of features maintained a high predictive power, while avoiding the risk of overfitting.

Overall, this study compared various machine learning classifiers, including Random Forest (RF) (33), Support Vector Machines (SVM) (34), Extra Trees (ET) (34), K-nearest neighbor (KNN) (35) and Mamba (36), ultimately selecting RF as the final classification model. The realization of multi-level fusion of DLR and clinical metabolic parameters features is one of the most important innovations of our study. Our feature selection process was initially applied to deep learning features and imaging radiomics features, respectively, to obtain their respective optimal feature subsets. Subsequently, these two types of features were combined with PET metabolic parameters (including SUVmax, SUVmean, etc.). Subsequently, these were used in clinical practice to construct a Complex model integrating multi-source information.

**Figure 3:**
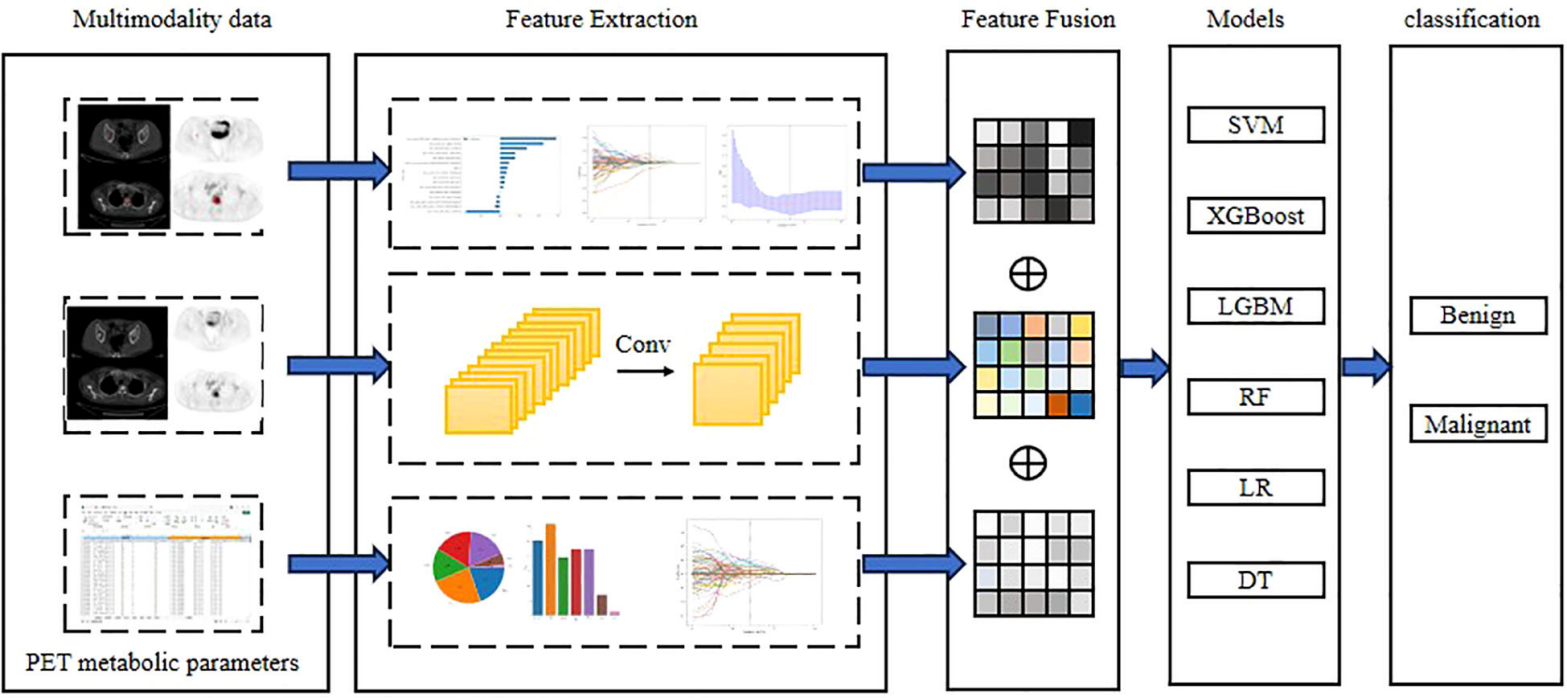
Workflow of Deep learning, Radiomics and Complex with PET metabolic parameters from multimodal data. Conventional radiomic features were extracted from CT, PET and PET/CT images. Feature selection and fusion techniques were applied to reduce dimensionality and integrate complementary information. BasicNet was employed in transfer learning using a pretrained model. The classiffcation model was constructed using six machine learning algorithm and visualisation of the decision process.

For the fusion strategy, feature-level, rather than decision-level, fusion was used. All selected features were combined into a single feature vector and input into the RF classifier. This strategy allowed the model to automatically learn the complex interactions between different types of features, and fully utilize the complementary advantages of each type of features. Experiments further demonstrated that the Complex model significantly outperformed models using only a single type of features (deep learning, radiomics, clinical parameter model), validating the effectiveness of multi-source feature fusion. The workflow for classification model construction is shown in **Figure 3**.

### 2.7 Statistical analysis

Statistical analysis was performed using SPSS version 26 (IBM Corp., USA) (37). Normally distributed data were expressed as mean ± standard deviation and compared using Student’s t-test. Non-normally distributed data were presented as medians and analyzed with the Mann–Whitney U test. Categorical variables were compared using the chi-square (χ²) test.Model performance was evaluated via ROC curves, sensitivity, specificity, accuracy, precision, and F1-score. The DeLong test was used to compare ROC curves between models, with P < 0.05 considered statistically significant.

## 3 Results

### 3.1 Clinical characteristics

A total of 207 female patients with clinically highly suspected breast cancer bone metastasis were enrolled, contributing 312 bone lesions, yielding an average of 1.5 lesions per patient. The mean age was 58.23 ± 14.05 years. Among the lesions, 107 were benign and 205 were malignant. The lesions were randomly divided into a training cohort (218 lesions) and a testing cohort (94 lesions).

**Figure 4:**
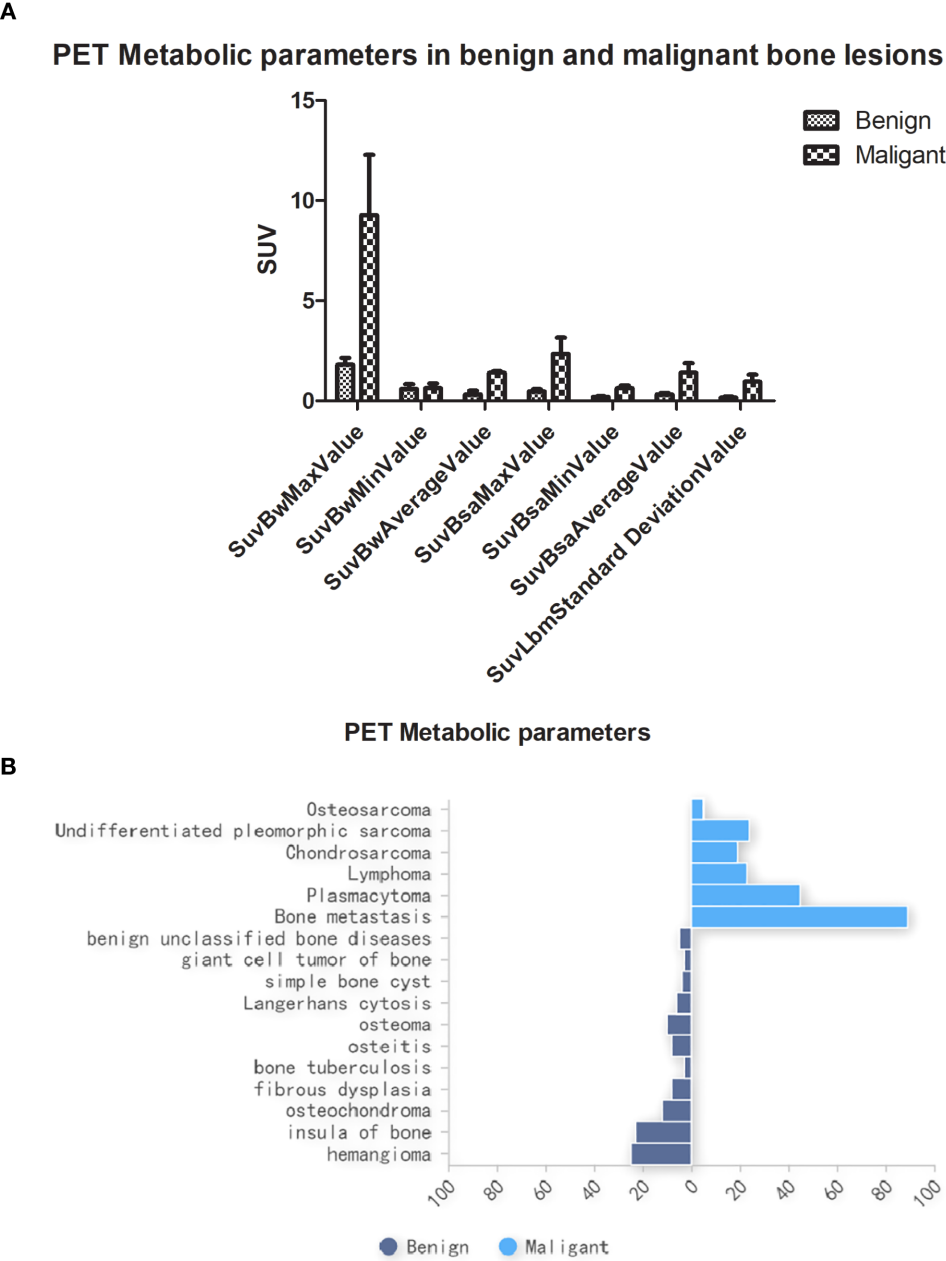
**(A)** PET metabolic parameters in benign and malignant bone oligolesions. **(B)** The pathology in benign and malignant bone oligolesions.

Clinicopathological and multimodal PET/CT imaging data yielded a total of 234,668 data features. Statistically significant differences (P < 0.05) were observed in PET metabolic parameters between benign and malignant bone lesions (**Figure 4A**). No significant differences were found in other clinical features across cohorts (P > 0.05), as detailed in **Table 1**. The pathological findings of the bone oligolesions are illustrated in **Figure 4B**.

**Table 1:** Characteristics of bone focals in breast cancer.

Characteristics	Training(n=218)	Testing(n=94)	Values (χ2)	*P*
Anatomical location			6.627	0.156
limbs	45(20.6%)	22(23.4%)	–	–
Vertebrae	57(26.1%)	19(20.2%)	–	–
Pelvis	49(22.5%)	27(28.7%)	–	–
Ribs	53(24.3%)	15(16.0%)	–	–
Skull	14(6.4%)	11(11.7%)	–	–
Bone structure changes			1.094	0.595
Osteolytic	87(39.9%)	33(35.1%)	–	–
Osteogenic	96(35.1%)	42(44.7%)	–	–
Mixed	35(38.5%)	19(20.2%)	–	–
Pathology			0.715	0.398
Benign	78(35.8%)	29(64.2%)	–	–
Malignant	140(30.9%)	65(69.1%)	–	–

### 3.2 Visual assessment based on PET/CT reader performance

A double-blind visual analysis was independently conducted by two experienced physicians (LGX and HSH, each with over 10 years of experience). Physicians were blinded to patients’ clinical and pathological data. Of the 312 lesions suspected of bone metastases, 98 (31.4%) showed morphological abnormalities on CT, 195 (62.5%) showed abnormalities in PET metabolic parameters, and 227 (72.8%) were abnormal on PET/CT fused images.

Through visual assessment, 57 lesions (53.3%) were diagnosed as benign and 138 (67.4%) as malignant. According to the reference standard, 89 of the malignant lesions (43.4%) were ultimately confirmed as bone oligometastases. The diagnostic accuracy of PET/CT was 50.6% (45/89).

### 3.3 Radiomics analysis of multimodal imaging

Rectangular ROI images were extracted from 48 groups of CT and PET feature values. Features were classified into the single-mode radiomics model and perfusion model (PET/CT) features. Overall, 7 classifiers were tested in the experiment to finally yield the optimal classification model. Notably, when training the first classification model, we set up random seeds to fix the instances of the training and test sets, to ensure the consistency of training and testing of all classification models and thus the fairness of model evaluation.

Multimodal fusion models combining CT, PET, and PET/CT images demonstrated high validity and stability. Diagnostic metrics for different imaging modalities were calculated for both the validation and test cohorts. Among the single modalities, PET radiomics achieved higher accuracy (97.8%) and AUC (0.970) compared to CT. The PET/CT fusion model had a sensitivity of 93.4% [80.3%–97.5%]. The best-performing model, a complex classifier, achieved a sensitivity of 96.1% [75.7%–99.4%], specificity of 98.2% [88.1%–99.6%], accuracy of 98.7% [89.6%–99.5%], and AUC of 0.989 [0.927–0.994], as shown in **Table 2**.

**Table 2:** Results of radiomic performance with conventional features.

Modality	AUC	Sensitivity	Specificity	Accuarcy	Classifiers
CT	0.970	0.868[0.793–0.897]	0.882[0.893–0.929]	0.939 [0.875–0.979]	SVM
PET	0.970	0.926[0.875–0.978]	0.947[0.812–0.973]	0.978 [0.817–0.983]	RF
PET/CT	0.980	0.934[0.803–0.975]	0.963[0.862–0.983]	0.981 [0.892–0.996]	XGBoost
Complex	0.987	0.961[0.757–0.994]	0.982[0.881–0.996]	0.989 [0.896–0.995]	LightGBM

# [] represents the 95% conffdence intervals (CI).

### 3.4 Deep learning models for multimodal imaging

A CNN-based segmentation model was developed using a 3D U-Net architecture. Input data included CT, PET, and label masks, which were resampled using trilinear interpolation and concatenated along the channel dimension. Each lesion patch had a volume of 100 × 100 × 100 voxels with a voxel size of 3.0 × 1.37 × 1.37 mm. The output of the CNN was a probability map for a 12 × 12 × 12 region at the center of the input patch.

Training employed categorical cross-entropy loss with evenly sampled training data from both classes. To improve learning, more samples were drawn from regions with high SUV uptake and previously misclassified voxels to emphasize difficult-to-classify areas (38–40). For single-modality models, PET achieved an accuracy of 94.7%, sensitivity of 90.4%, and specificity of 95.9%. The multimodal fusion model demonstrated an improved accuracy of 96.3%, with an AUC of 0.979, sensitivity of 97.5% [85.7%–98.7%], and specificity of 90.3% [81.9%–92.5%]. The ensemble fusion model further enhanced performance with an accuracy of 97.0%, AUC of 0.986, sensitivity of 93.2%, and specificity of 91.6%, as summarized in **Table 3**.

**Table 3:** Results of transfer learning classiffcation utilising deep features.

Modality	AUC	Sensitivity	Specificity	Accuarcy	Classifiers
CT	0.960	0.833[0.784–0.881]	0.880[0.781–0.946]	0.956 [0.812–0.976]	SVM
PET	0.980	0.904[0.860–0.984]	0.959[0.863–0.985]	0.947 [0.881–0.982]	ExtraTrees
PET/CT	0.979	0.975[0.857–0.987]	0.903[0.819–0.925]	0.963[0.916–0.972]	RF
Complex	0.986	0.932[0.884–0.965]	0.916[0.835–0.967]	0.970[0.832–0.979]	SVM+KNN

# [] represents the 95% conffdence intervals (CI).

### 3.5 DLR fusion models

To further improve classification performance, conventional radiomics features were integrated with deep learning (DL) features derived from multimodal imaging of bone lesions of breast cancer oligometastases. Feature fusion of DLR features was performed using Z-score normalization. The Spearman correlation coefficient was used to assess feature correlations, retaining one feature from pairs with a correlation above 0.9. LASSO logistic regression was subsequently applied, with penalty parameter tuning via 10-fold cross validation to identify bone lesions features with nonzero coefficients (41). The classification model combining radiomics and DL features showed robust performance.

Evaluation of the performance of the PET/CT fusion models revealed that the complex ensemble model with PET/CT fused clinical parameters in RF classifier achieved the best AUC of 0.990, as well as the highest accuracy, sensitivity, specificity and accuracy of 98.6%, 99.8%, and 99.7%, respectively (**Table 4**).

**Table 4:** The feature fusion results of conventional radiomic features and deep features from transfer learning.

Methods	AUC	Sensitivity	Specificity	Accuracy	Classifier
Basic Net	0.985	0.985 [0.818-0.993]	0.965 [0.894-0.985]	0.970 [0.884-0.996]	SVM
ResNet-101	0.786	0.768 [0.660-0.842]	0.718 [0.631-0.775]	0.774 [0.619-0.795]	KNN
DCU-Net	0.814	0.895 [0.754-0.908]	0.712 [0.683-0.772]	0.794 [0.730-0.837]	Mamba
S-Net	0.807	0.871 [0.716-0.912]	0.642 [0.584-0.716]	0.743 [0.695-0.810]	ExtraTrees
DLR	0.954	0.937 [0.861-0.992]	0.925 [0.868-0.969]	0.957 [0.892-0.979]	RF
DLR+Complex	0.990	0.986 [0.896-1.000]	0.998 [0.905-1.000]	0.997 [0.917-0.999]	RF

DLR represents fused deep learning radiomics.

### 3.6 Comparison of classification models

**Figure 5:**
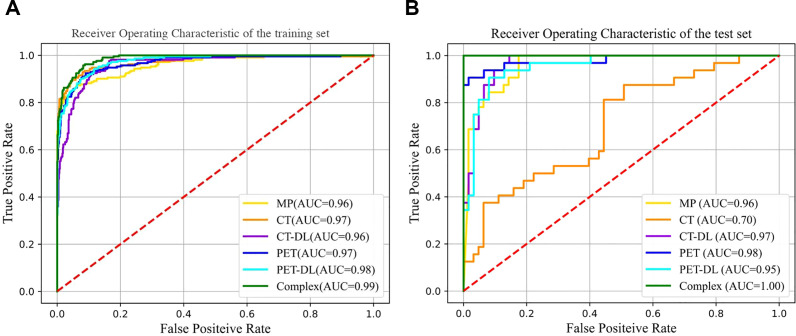
Comparison of ROC curves under different classiffers with trainging set **(A)** and testing set **(B)**.

Various classification models were compared to distinguish benign from malignant bone oligolesions in breast cancer. Models based on BasicNet (24), ResNet-101 (42), DCU-Net (43), and S-Net (44) were evaluated. The best performance was achieved by the complex model that combined traditional radiomics, DL, and clinical parameters using the RF classifier. This model performed best in both the training and testing cohorts. The comparative results are illustrated in **Figures 5A, B**.

The DLR+Complex model showed the best performance on all evaluation metrics, with an AUC of 0.990, sensitivity of 98.6% [95% CI: 0.896-1.000], specificity of 99.8% [95% CI: 0.905-1.000], and accuracy of 99.7% [95% CI: 0.917-0.999]. More importantly, compared with the BasicNet model, the AUC of the DLR+Complex model increased by 0.005 and the accuracy increased by 0.27, demonstrating a more significant performance advantage. Further performance comparison analysis shows that compared with the BasicNet model, the DLR+Complex model has improved sensitivity (98.6% vs 98.5%), specificity (99.8% vs 96.5%), and accuracy (99.7% vs 97%), which is of great significance in clinical applications.

## 4 Discussion

Bone is the third most common site of metastasis after the lungs and liver. Bone metastases most frequently originate from breast and prostate cancers, which together account for approximately 70% of primary tumors (45). Bone is affected by various types of malignancies. Among them, bone oligometastases of breast cancer represents a significant subtype, for which early diagnosis and precision treatment are critical for improving patient outcomes. However, the unique biological characteristics of bone oligometastases still require further investigation, especially the roles played by microenvironmental remodeling and the osteogenic/osteoclastic balance, both of which are crucial in determining prognosis (46).

The most commonly used diagnostic modality for detecting bone metastases is whole-body bone scintigraphy (WBS) using ^99m^Tc-MDP due to its high sensitivity and full-body scanning capability (47). However, WBS has limited accuracy in detecting osteoclastic lesions, particularly when the number of lesions is fewer than three or the lesion size is under 1 cm.

Our study offers significant advancements by integrating radiomics and DL models with multimodal PET/CT images to improve the prediction of bone metastasis in breast cancer. Based on expert diagnostic visual assessment, CT scans detected 98 abnormal morphological lesions—56 osteogenic, 29 osteoclastic, and 13 mixed-type. However, CT alone failed to identify 214 lesions, demonstrating its limitations as a single modality.

In contrast, PET/CT provides a multidimensional view by combining metabolic and anatomical information, which is critical for early diagnosis, disease staging, and evaluating treatment efficacy. Fused PET/CT images revealed 227 abnormal foci, including 89 osteoclastic lesions. The diagnostic accuracy of visual evaluation in distinguishing benign from malignant lesions was 53.3% and 67.4%, respectively. Using a conventional radiomics model, fused PET/CT imaging achieved improved diagnostic performance, with an accuracy of 90.1%, specificity of 86.3%, sensitivity of 83.4%, and an AUC of 0.894.

Pallavi et al. (32) also found that combined models incorporating PET and contrast-enhanced CT (CECT) outperformed single-modality models in differentiating multiple myeloma from skeletal metastases. Other studies have similarly reported positive outcomes using radiomics and traditional machine learning techniques for bone metastasis prediction. For example, Chen et al. (48) demonstrated the effectiveness of a Vision Transformer model, which achieved an AUC of 0.918 on the test set in predicting bone metastasis in colorectal cancer using both plain and contrast-enhanced CT. Song (49) also developed a semi-automated model integrating radiomics, DL, and clinical features using biparametric MRI, achieving an internal AUC of 0.934 and an external AUC of 0.903 for bone metastasis prediction in prostate cancer.

Our results are consistent with the above findings, confirming the value of radiomics features in predicting metastasis. Meanwhile, we compared the Mamba-based recent classifier with the traditional classifies.With its unique structure and algorithmic optimization, the Mamba with multimodal feature fusion method can efficiently extract and fuse the features of different modalities to enhance the system performance and efficiency (50). Its high efficiency and accuracy have made it an important strategy for multimodal data processing (9, 51).While in our study, although the former based on generative feature extraction effectively integrates the datas from CT and PET, it only achieved good performance in the DCU-net model, while the other models underperformed, which may be related to less raw datas in our study.

**Figure 6:**
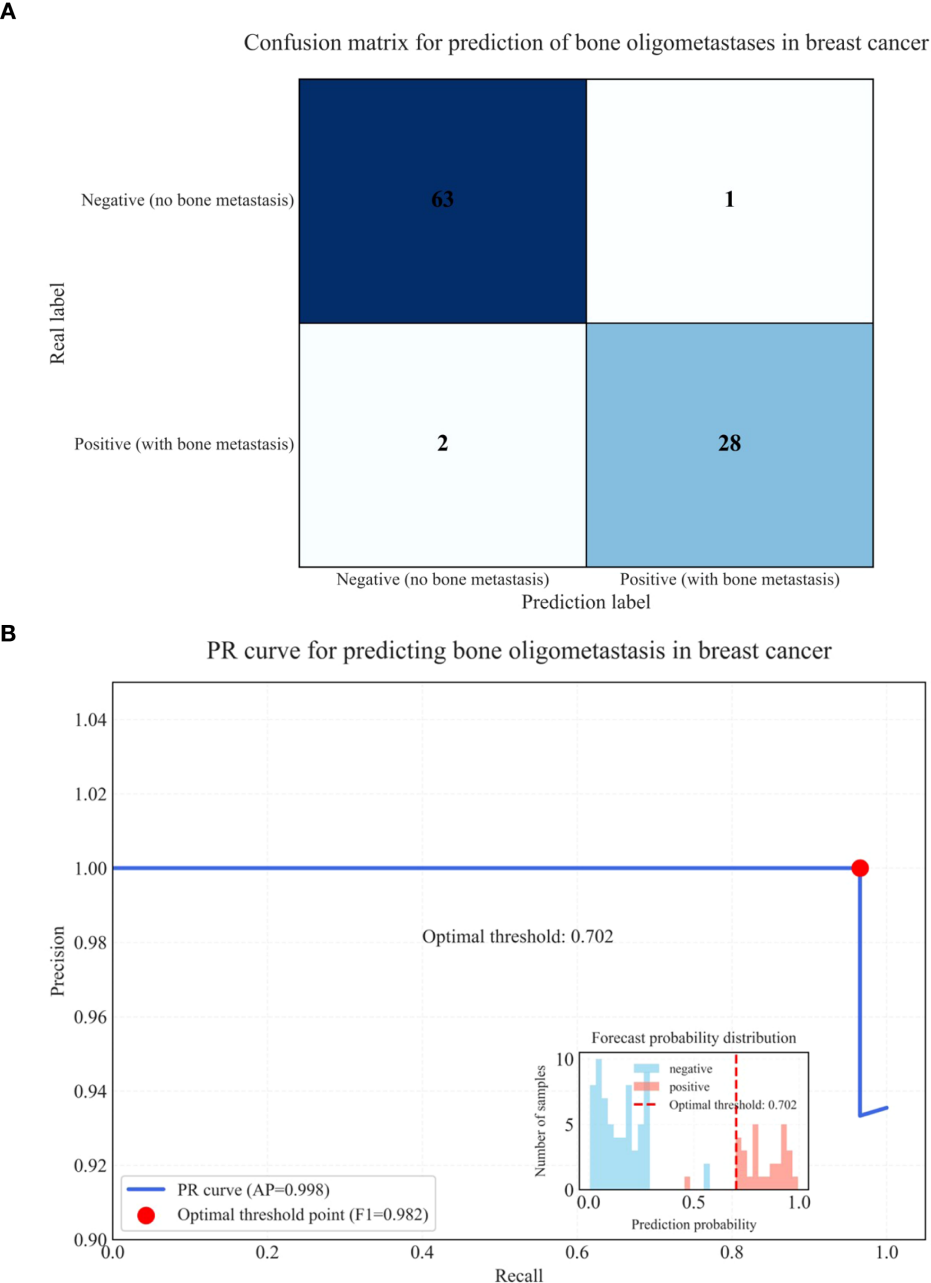
Confusion matrix for prediction of bone oligometastases in breast cancer **(A)** and PR curve for predicting bone oligometastases in breast cancer **(B)**.

Few studies have specifically targeted bone oligometastases in breast cancer using PET/CT with integrated DLR. In our study, the integration of radiomics, DL (particularly the BasicNet model), and clinical metabolic parameters significantly enhanced predictive performance. The DLR + Complex model achieved outstanding diagnostic metrics, including an AUC of 0.990, accuracy of 99.7%, specificity of 99.8%, and sensitivity of 98.6%, indicating exceptional discriminative ability and generalizability.Our model’s superior performance may stem from its ability to automatically learn complex features directly from raw CT, PET, and fused PET/CT images, combined with clinical metabolic parameters. DL methods capture intricate spatial relationships and hierarchical patterns. These architectural advantages—such as enhanced feature reuse and improved gradient flow—contributed to the model’s high accuracy and robustness. The DLR + Complex model based on multimodal PET/CT imaging has significant potential in accurately identifying and monitoring bone oligometastases in breast cancer, thereby enabling timely and individualized treatment strategies. The confusion matrix (28 cases of true positive, 1 case of false negative, 2 cases of false positive, 63 cases of true negative) reveals the core performance characteristics of the model in the prediction of bone oligometastasis of breast cancer, as shown in (**Figure 6A**). From a clinical perspective, the high sensitivity (98.6%) showed that the model had excellent positive case capture ability, and only one false negative case was missed. This is crucial in the screening of metastatic cancer, because missed diagnosis may lead to treatment delay. The high specificity (99.8%) proved that the model can effectively exclude non metastatic patients and avoid over treatment. Combined with the overall accuracy of 98.6%, it was confirmed that the deep learning radiomics model was significantly superior to the conventional diagnostic method. The deep learning radiomics model established in this study has achieved effective improvement in sensitivity, specificity and accuracy, and its PR curve has confirmed its stability under high recall demand, providing a reliable tool for early intervention of bone metastasis of breast cancer, as shown in (**Figure 6B**).

Despite our promising results, several limitations remain, as follows:

1. The sample size of 312 lesions is relatively small and lacks diversity in oligometastases types (52).
2. The study is a single-center, retrospective design, which may introduce selection bias and does not encompass all subtypes and metastatic patterns of breast cancer (53).
3. The DLR + clinical complex models are still in the experimental stage and require prospective, multicenter validation.
4. This research focused solely on the diagnostic performance of PET/CT images and did not investigate the relationship between imaging features and underlying molecular mechanisms, such as bone microenvironmental remodeling.

Future research should therefore aim to expand the sample size, improve automated analysis pipelines, and integrate multi-omics data with imaging features. These efforts will support the advancement of precision diagnostics and therapeutic strategies for breast cancer patients with bone oligometastases.

## 5 Conclusion

Overall, this study demonstrates the potential of integrating radiomics, DL, and clinical complex models to predict bone oligometastases in breast cancer patients using fused PET/CT imaging. The DLR + Complex model significantly outperformed traditional radiomics and other deep learning architectures, achieving high AUC, accuracy, specificity, and sensitivity in both training and testing cohorts.

Accurate early prediction of bone oligometastases enables timely, targeted treatment interventions, improving patient outcomes and optimizing resource utilization. Ultimately, while PET/CT-based models show strong predictive power, further refinement incorporating histological subtypes, imaging features, and molecular biomarkers will be essential for comprehensive and personalized diagnosis.

Taking histological subtypes as an example, based on our data analysis and relevant literature, triple negative breast cancer (TNBC) and HER2 positive breast cancer showed a higher tendency of bone metastasis. Specifically, the risk of bone metastasis in TNBC patients is about 2–3 times higher than in hormone receptor positive patients, and the metabolic activity of metastatic lesions is higher, which is manifested as higher SUVmax values in our PET/CT imaging. HER2 positive breast cancer tends to have multiple bone metastases rather than oligo metastasis. Therefore, future predictive models should consider these molecular subtypes as key stratification factors, especially in TNBC and HER2 positive patients, which may require the development of more sensitive early detection strategies.

## Data Availability

The original contributions presented in the study are included in the article/supplementary material. Further inquiries can be directed to the corresponding authors.
